# On the Detection and Use of Alcohol

**Published:** 1863-05

**Authors:** Edward Smith


### ON THE DETECTION AND USE OF ALCOHOL.

BY EDWARD SMITH, M. D.

From the British Medical Journal, November 2d and 16th.

The following is a summary of the author's views on the action of alcohol upon the system:

1. It increases the force of the heart's action, as proved by the fulness and sharpness of the pulse, and by the pulsation at the temples, and in the small arteries of the hands as they lie upon the table, and the height to which the foot is jerked when, after taking a glass of spirit and water, we sit with the legs crossed.

2. It determines the blood to the extremities and to the surface, the latter fact being proved by the increased redness of the hands and face, with which all are familiar. This we presume to be due to the first action.

3. It lessens, or tends to lessen, the action of the skin, as proved by the effects of rum and alcohol in the experiments, by the state of skin in those with whom it has disagreed, whether in health or disease, and by the ordinary condition of the skin in persons who habitually drink much alcohol. The action of the skin *par excellence* is the elimination of vapor, and when this action is lessened the skin becomes dry. It is not affirmed that the skin always becomes dry under the influence of alcohol; but that it commonly does so, and that as such is its action, when taken in excess, such will necessarily be the direction of its action when taken in a less degree.

4. It increases the heat of the skin. This will follow from the three former statements; for if more warm blood be sent to a surface in contact with air, and which, therefore, must, at ordinary temperatures, be cooler than the blood, there will be an addition to its heat; and also, if the vaporization by the skin be lessened, and by it the abstraction of heat from the surface in the supply of the latent heat when a fluid is converted into vapor, as in the perpetual action of the skin, be lessened, it follows that, with a less dispersion of heat and an increased supply of heat, the skin will become hotter. This is based upon physical laws which cannot be controverted, and accounts for the sense of increased heat, without in any way affecting the question of increased production of heat, by the metamorphosis of the alcohol.

5. That alcohol renders the hands and face red, hot, and swollen, is matter of common observation.

6. When alcohol is taken in a moderate state of dilution, it produces a sensation of heat in the mouth, throat, and stomach; and, from what we know of the action of various stimulants to the mucous surface, it will increase the action of the surface if diluted, or it will arrest the action if it be concentrated, and in this respect its action is doubtless analogous to that of warm or hot water.

7. It lessens excretion of water by the kidneys. This has been asserted by all experimenters, as Vierordt, Böcker, Hammond, and Smith; and in the author's recent experiments in prisons, he found the urine was reduced twenty ounces per day on the average of three days by two ounces of alcohol daily, in four prisoners working the treadwheel, and who for months had not taken alcohol. Some alcohols, as gin, increase the action of the kidneys, at least, for a time; but that action is not due to the alcohol.—When alcohol *appears* to do so, it is by lessening the powers of the sphincters.

8. It lessens the excretion of urea by the kidneys. This also has been universally admitted, and will follow from the diminished excretion of urine.

9. It lessens the excretion of chloride of sodium in proportion to the diminution in the quantity of urine.

10. It lessens the excretion of fæces, as has been observed by all experimenters, and particularly by Hammond and the author; and this is apparently due to the withdrawal of fluid from the bowel; for when the fæces are passed, they are unusually hard, and passed with some difficulty.

11. If there be less fluid emitted under the action of alcohol by the skin, kidneys and bowels, and the quantity of fluid introduced into the body be not lessened, does it not follow that alcohol has the power of causing the secretion of an increased quantity of fluid in the tissues, and thus of increased bulk and weight? What is meant by the popular observation that a person rapidly fed up and bloated by alcohol has not sound fat, but that the rapidity with which it often passes away proves that the bulk is chiefly due to fluid?

12. These effects occur during a limited period, since the period of the duration of all actions is limited; and after one hour and a half the force of the heart subsides; in less than an hour the sensation of heat diminishes; after several hours, the skin regains its normal degree of action; and after one or more days, the normal excretion by the kidneys and bowels is re-established.

13. We need not refer to the action of alcohol in lessening consciousness, the perception of light and sound, and the diminution of muscular power; for when a full ordinary dose is taken, they may be perceived in every half-drunken man; and Smith has already minutely described the period of this occurrence in experiments upon himself and others. In less doses, these effects are either less evident or they are not at all perceptible; but in whatever dose, the direction of the action of the alcohol must be the same. It is impossible that a small dose of alcohol shall *directly* increase muscular power, for example, whilst a greater yet an ordinary dose decreases it; and if men half drunk have sometimes exerted unusual strength, it has been from the same cause as is seen in the efforts of a madman, not from increase of muscular power, but from increase of the effort of the will.

